# Prevalence of Municipal-Level Policies Dedicated to Transportation That Consider Food Access

**DOI:** 10.5888/pcd18.210193

**Published:** 2021-11-18

**Authors:** Brianna L. Dumas, Diane M. Harris, Jean M. McMahon, Thomas J. Daymude, Amy Lowry Warnock, Latetia V. Moore, Stephen J. Onufrak

**Affiliations:** 1 National Center for Chronic Disease Prevention and Health Promotion, Centers for Disease Control and Prevention, Atlanta, Georgia; 2 Center for Preparedness and Response, Centers for Disease Control and Prevention, Atlanta, Georgia; 3 National Center for Emerging and Zoonotic Infectious Diseases, Centers for Disease Control and Prevention, Atlanta, Georgia

## Abstract

**Introduction:**

Local governments can address access to healthy food and transportation through policy and planning. This study is the first to examine municipal-level transportation supports for food access.

**Methods:**

We used a nationally representative sample of US municipalities with 1,000 or more persons from the 2014 National Survey of Community-Based Policy and Environmental Supports for Healthy Eating and Active Living (N = 2,029) to assess 3 outcomes: public transit availability, consideration of food access in transportation planning, and presence of demand-responsive transportation (DRT). We used χ^2^ tests to compare prevalences by municipal characteristics including population size, rurality, census region, median educational attainment, poverty prevalence, racial and ethnic population distribution, and low-income low-access to food (LILA) status.

**Results:**

Among municipalities, 33.7% reported no public transit and 14.8% reported having DRT. Both public transit and DRT differed by population size (both *P* < .001) and census region (both *P* < .001) and were least commonly reported among municipalities with populations less than 2,500 (46.9% without public transit; 6.6% with DRT) and in the South (40.0% without public transit; 11.1% with DRT). Of those with public transit, 33.8% considered food access in transportation planning; this was more common with greater population size (55.9% among municipalities of ≥50,000 persons vs 16.8% among municipalities of <2,500 persons; *P* < .001), in the West (43.1% vs 26.8% in the Northeast, 33.7% in the Midwest, 32.2% in the South; *P* = .003), and municipalities with 20% or more of the population living below federal poverty guidelines (37.4% vs 32.2% among municipalities with less than 20% living in poverty; *P* = .07).

**Conclusion:**

Results suggest that opportunities exist to improve food access through transportation, especially in smaller and Southern communities, which may improve diet quality and reduce chronic disease.

**Summary**

**What is already known on this topic?**

Local governments can address access to food and transportation through policy and planning.

**What is added by this report?**

We assessed data from municipalities regarding food and transportation from the 2014 National Survey of Community-Based Policy and Environmental Supports for Healthy Eating and Active Living. One-third of municipalities reported not having public transit, and 14.8% reported having demand-responsive transportation, with differences by municipal-level characteristics. Of those with public transit, 33.8% considered food access in transportation planning.

**What are the implications for public health practice?**

Results suggest an opportunity to improve food access by using public transportation supports, especially in communities that have small populations, are rural, and are in the South.

## Introduction

According to 2017–2018 data from the National Health and Nutrition Examination Survey (NHANES), the age-adjusted prevalence of obesity in adults is 42.4% (1). To address the complex etiology of obesity and diet-related chronic disease, cross-disciplinary strategies are required. The Centers for Disease Control and Prevention (CDC) (2), the Institute of Medicine (3), and the American Heart Association (4) have recognized food access as an important strategy to reduce the overall prevalence of obesity and improve public health. Nevertheless, food access is still an issue across the US. The US Department of Agriculture (USDA) estimates that 40 million Americans live in communities with poor access to food retailers such as supermarkets, and this burden is most heavily concentrated in rural, low-income, and racial and ethnic minority communities (5).

Even where supermarkets, large grocery stores, and farmers markets are present in a community, some people may still have difficulty obtaining foods because of resource constraints or proximity to food outlets. For example, 2.1 million US households do not own an automobile and are 20 miles away from a supermarket, with the lowest rates of car ownership being among low-income communities and communities of color (5,6). Public transit can provide affordable mobility to access basic needs such as medical care and food, especially for those who do not own a car or are physically impaired or immobile. Connecting routes such as sidewalks, trails, and public transit to everyday destinations such as grocery stores can also improve physical activity (7). Furthermore, there is emerging evidence to support public transit use as an intervention strategy to control obesity (8–11). Despite a 21% overall increase in public transit ridership since 1997 (6), 45% of US households still do not have access to public transit and almost 20% of people in the US experience significant transportation barriers to accessing healthy foods (12). In rural communities, lack of transportation infrastructure is regarded as the largest and most acute obstacle to accessing food (11). Providing and improving transportation options to and from food retail locations, in addition to in-store and community food programs (eg, fruit and vegetable prescription programs) increases community members’ overall access to food. For example, demand-responsive transit (DRT), which is a form of private or quasi-public transportation that provides more individualized rides by using smaller buses or vans without fixed routes or timetables, can be used to provide transportation to food retailers as an alternative to fixed-route public transit systems or in areas without public transit systems (13).

Local governments can address community needs such as food access and transportation through policy and planning. The purpose of this study was to examine the extent to which food access is considered in municipal-level transportation policy and planning in a nationally representative sample of US municipalities with 1,000 or more persons. Specifically, this study examined the percentage of municipalities that lack public transit, the percentage that consider food retail accessibility in their public transit planning, and the percentage that offer DRT transportation to food retailers. We also examined whether the prevalence of these policies differed according to municipal characteristics such as size, region, and low-income low-access (LILA) status (formerly known as “food desert”). To our knowledge, no large-scale studies have examined the prevalence of municipal-level policies that support transportation to access foods.

## Methods

Our cross-sectional study used data from the National Survey of Community-Based Policy and Environmental Supports for Healthy Eating and Active Living (CBS HEAL) survey. The purpose of the CBS HEAL survey was to collect information on existing policies and practices, enacted or implemented by local governments, that promote healthy eating and physical activity. The survey had 3 modules that consisted of questions that underwent cognitive testing and were piloted in 2 states (14). The survey was offered online with an option of completing a paper version; it was sent to city or town managers, or positions with similar job duties or responsibilities. Respondents were encouraged to ask other municipal officials for help completing the survey. Participation was voluntary but for those who participated, CDC shared a benchmark comparison report that showed how their municipality compared with others.

The CBS HEAL survey was conducted by the Division of Nutrition, Physical Activity, and Obesity at CDC from May through September 2014. The sampling frame was based on the US Census Bureau’s 2007 Census of Governments (15), which lists municipal governments (municipalities) by state and was the most current data available at the time the sample was selected. Results from the aforementioned pilot found municipalities with fewer than 1,000 persons were less likely to have these policies, thus were excluded from the sample pool. The final sample was 4,484 municipalities from a possible 10,205 eligible municipalities with 1,000 or more persons. Municipalities were sampled stratified by census region and rurality and sorted by population size to generate a nationally representative sample. Sample weights were developed to allow for more nationally representative estimates and accounted for the sampling methodology and nonresponse. A total of 2,029 municipalities returned the survey for a response rate of 45%. For our study, 10 municipalities were excluded who did not respond to the 2 primary study questions regarding transportation supports for food accessibility.

This study examined 3 outcome variables: 1) availability of public transit, 2) consideration of food access when routing public transit, and 3) availability of DRT to food retail locations. Availability of public transit and consideration of food access when routing public transit were determined by using the survey question “Does your local government consider accessibility to supermarkets or other full-service grocery stores in their assessment of public transportation routes?” (Response options: yes, no, don’t know, and “our community does not have public transportation”). Municipalities that answered yes, no, or don’t know were classified as having public transit available while those that answered “our community does not have public transportation” were classified as not having public transit. Classification of consideration of food accessibility when routing public transit was assessed only among municipalities that answered yes, no, and don’t know. Municipalities that answered yes were classified as municipalities that consider food access when routing public transit; municipalities that answered no or don’t know were classified as those that do not consider food access when routing public transit.

Availability of DRT to food retail was determined by using the question “Does your local government have a policy that supports dedicated transportation (eg, community vans or shuttle buses) to supermarkets, other full-service grocery stores, or farmers markets for these residents? Do not include public transportation options in your response.” Response options included yes, no, and don’t know. Municipalities that responded yes were classified as having DRT while those that responded no or don’t know were classified as not having it. Because this survey question instructed respondents not to include public transportation in their response, the prevalence of DRT includes municipalities that may or may not have public transit.

Municipal characteristics used as covariates in the analysis included population size (<2,500, 2,500–49,999, or ≥50,000 persons), rural/urban status (rural or urban), census region (Northeast, Midwest, South, or West), median educational attainment among those aged 25 years or older (some college or more or high school graduate or less), poverty prevalence (<20% or ≥20% of the population living below federal poverty guidelines), racial and ethnic population distribution (>50% non-Hispanic white or ≤50% non-Hispanic white), and LILA status (≥1 LILA census tracts or no LILA census tracts). Municipal population size was obtained from the 2007 Census of Governments (15). Rural/urban status was derived from the 2010 US Census Urban Area to Place Relationship File, with municipalities classified as urban if more than 50% of the population resided in areas defined as urban (16). Data from the 2009–2013 American Community Survey were used to determine median education level of the population, racial and ethnic population distribution, and percentage of the population living below the federal poverty guidelines (17). For the poverty rate variable, the cut point of 20% was based on the definition of persistent poverty used by USDA (18). LILA status was determined by geographically matching municipal geographic boundaries intersecting with LILA census tracts from the USDA Food Access Research Atlas (19). A LILA tract is defined by USDA as “a low-income tract with at least 500 people, or 33 percent of the population, living more than 1 mile (urban areas) or more than 10 miles (rural areas) from the nearest supermarket, supercenter, or large grocery store” (20). LILA status could not be determined for 71 municipalities where the municipality could not be matched to census tracts (because of census tract changes between the 2000 and 2010 censuses) or where the municipality linked to a single census tract that overlapped more than 5 municipalities, because the LILA status of a census tract containing many municipalities may not necessarily apply to all the municipalities.

The weighted prevalence and associated 95% CIs of not having public transit available and having DRT to food retail were calculated overall and according to municipal characteristics. The prevalence and associated 95% CIs of planning public transit routes to support food accessibility were estimated both overall and according to municipality characteristics among the municipalities where public transit was available. We used χ^2^ tests to determine whether prevalence differed according to municipal characteristics, with *P* values less than .05 determined to be significant. Multivariable logistic regression models were then fit to calculate odds ratios and 95% CIs to assess the independent relation of municipal characteristics with each outcome. Associations were determined to be significant if the CI did not include 1.0. Because 71 municipalities were missing data on LILA status, 2 models were fit for each outcome. The first series of models included those with missing information on LILA status and estimated odds ratios between the outcome with population size, rural/urban status, census region, median educational attainment, poverty prevalence, and racial and ethnic population distribution, controlling for the other variables. The second series of models excluded those with missing information of LILA status and estimated the odds ratio between the outcome and LILA status while controlling for the remaining variables among municipalities for which we were able to ascertain LILA status. To assess the extent to which don’t know responses may have affected the results, we also performed sensitivity analyses where we recalculated prevalences of transportation supports and their relationship to municipal characteristics, excluding respondents who answered don’t know to each transportation question.

## Results

Approximately one-third of municipalities reported that they did not have public transit (Table 1). Availability of public transit differed according to population size, rural/urban status, census region, racial and ethnic population distribution, and LILA status. Nearly half (46.9%; 95% CI, 43.2%–50.5%) of municipalities with fewer than 2,500 persons reported that they did not have public transit compared with 29.2% (95% CI, 26.5%–31.8%) of those with 2,500 to 49,999 persons, and only 5% (95% CI, 1.4%–8.6%) of those with 50,000 or more persons. Rural municipalities more commonly lacked public transit than urban municipalities (50.4% vs 28.0%, *P* < .001). Not having public transit was most common among Southern municipalities (40.0%) and least common in the West (16.3%). Municipalities where median education attainment was high school graduate or less more commonly reported no public transit compared with municipalities where median educational attainment was at least some college (39.4% vs 29.1%, *P* < .001). Lack of public transit was also more prevalent among municipalities with more than 50% non-Hispanic white population (34.7% vs 27.1% among municipalities with 50% or less non-Hispanic white, *P* < .01). Lacking public transit was also more common among municipalities that had no LILA designation (37.3% vs 25.7% among municipalities with LILA tracts, *P* < .001). In multivariate models, smaller population size, rural status, being in the Southern census region, and greater poverty prevalence were all significantly associated with lack of public transit. Median educational attainment, racial and ethnic profile, and LILA status were not significantly associated with presence of public transit.

**Table 1:** Lack of Public Transit and Availability of Demand-Responsive Transit to Support Food Access Among US Municipalities by Municipality Characteristics, National Survey of Community-Based Policy and Environmental Supports for Healthy Eating and Active Living, 2014

Municipality Characteristics	Municipality Does Not Have Public Transit			Municipality Has Dedicated Transportation to Food Retail Locations (eg, Community Vans or Shuttle Buses)		
	% Reporting No Public Transit (95% CI)	χ^2^ *P* Value	Odds Ratio^a^ (95% CI)	% Reporting Yes (95% CI)	χ^2^ *P* Value	Odds Ratio^a^ (95% CI)
**Overall** (n = 2,019)	33.7 (31.7–35.7)	Not applicable		14.8 (13.2–16.4)	Not applicable	
**Population**						
<2,500 (n = 719)	46.9 (43.2–50.5)	<.001	1 [Reference]	6.6 (4.8–8.4)	<.001	1 [Reference]
2,500–49,999 (n = 1,158)	29.2 (26.5–31.8)		0.7 (0.5–0.9)	19.2 (16.9–21.5)		4.0 (2.6–6.4)
≥50,000 (n = 142)	5.0 (1.4–8.6)		0.1 (0.05–0.2)	19.1 (12.6–25.6)		4.1 (2.1–7.7)
**Rural/urban status^b^**						
Urban (n = 1,480)	28.0 (25.7–30.3)	<.001	1 [Reference]	17.2 (15.2–19.1)	<.001	1 [Reference]
Rural (n = 539)	50.4 (46.2–54.6)		1.6 (1.2–2.1)	7.9 (5.6–10.1)		1.2 (0.8–2.0)
**Census region**						
Northeast (n = 234)	27.9 (22.2–33.6)	<.001	0.5 (0.4–0.8)	20.6 (15.4–25.8)	<.001	2.2 (1.5–3.4)
Midwest (n = 745)	36.7 (33.3–40.0)		0.7 (0.6–0.9)	14.9 (12.3–17.4)		1.6 (1.1–2.2)
South (n = 706)	40.0 (36.4–43.6)		1 [Reference]	11.1 (8.8–13.4)		1 [Reference]
West (n = 334)	16.3 (12.5–20.1)		0.3 (0.2–0.5)	18.0 (13.9–22.2)		1.7 (1.2–2.5)
**Median educational attainment^c^**						
Some college or more (n = 1,127)	29.1 (26.4–31.7)	<.001	1 [Reference]	15.3 (13.2–17.5)	0.5	1 [Reference]
High school graduate or less (n = 892)	39.4 (36.3–42.6)		1.3 (1.0–1.6)	14.1 (11.8–16.4)		1.3 (0.9–1.7)
**Poverty prevalence^d^**						
<20% (n = 1,407)	34.3 (31.9–36.8)	<.4	1 [Reference]	15.4 (13.5–17.3)	.3	1 [Reference]
≥20% (n = 612)	32.2 (28.5–35.9)		0.7 (0.5–0.9)	13.5 (10.8–16.2)		1.0 (0.7–1.3)
**Race and ethnicity**						
>50% Non-Hispanic White (n = 1,751)	34.7 (32.5–36.9)	<.01	1 [Reference]	14.6 (12.9–16.3)	.5	1 [Reference]
≤50% Non-Hispanic White (n = 268)	27.1 (21.8–32.4)		0.9 (0.6–1.2)	16.1 (11.7–20.6)		0.9 (0.6–1.1)
**Low-income low-access (LILA) status^e^**						
Contains LILA tracts (n = 621)	25.7 (22.3–29.2)	<.001	0.9 (0.7–1.1)	15.9 (13.0–18.8)	0.3	0.8 (0.6–1.1)
Does not contain LILA tracts (n = 1,327)	37.3 (34.8–40.0)		1 [Reference]	14.0 (12.1–15.9)		1 [Reference]

a Adjusted for population size, rural/urban status, census region, educational attainment, poverty prevalence, and race and ethnicity.

b Rural/urban status was derived from the 2010 US Census Urban Area to Place Relationship File, with municipalities classified as urban if more than 50% of the population resided in areas defined as urban (16).

c Among the population aged 25 years or older.

d Percentage of the population living below federal poverty guidelines.

e Model 1 includes all the municipalities and examines the associations for all the variables except the LILA variable; model 2 is used for the LILA variable and only includes municipalities with that variable.

The overall prevalence of DRT (eg, community vans or shuttle buses) to food retail was 14.8% (95% CI, 13.2%–16.4%). Less than 6% (5.9%) of municipalities without public transit reported that they had DRT services available, and 31.7% of municipalities had neither public transit nor DRT options available. Presence of DRT differed significantly by population size, rural/urban status, and census region (Table 1). Dedicated transportation services were more commonly reported by larger municipalities (19.1% among municipalities of ≥50,000 persons and 19.2% among municipalities of 2,500–49,999 persons vs 6.6% among municipalities with <2,500 persons, *P* < .001). Dedicated transportation services were also more commonly reported by urban compared with rural municipalities (17.2% vs 7.9%, *P* < .001) and varied by region (11.1%–20.6%, *P* < .001). In multivariable models, population size and census region were significantly associated with presence of DRT.

Among municipalities that had public transit (n = 1,338), approximately one-third (33.8%; 95% CI, 31.3%–36.4%) reported that they considered food retail accessibility in assessment of public transit routes and the prevalence differed according to population size, rural/urban status, census region, racial and ethnic population profile, and LILA status (Table 2). Prevalence increased with increasing population size, from 16.8% among municipalities with fewer than 2,500 persons to 55.9% among those with 50,000 or more persons (overall χ^2^ *P* < .001). Food retail accessibility in transit planning was also more commonly reported among urban municipalities (37.1% vs 19.6% among rural municipalities, *P* < .001). It was also most common among Western municipalities and least common among those in the Northeast (43.1 vs 26.8%, overall χ^2^ *P* = .003). Prevalence was also greater among municipalities with 50% or less non-Hispanic White persons compared with municipalities with more than 50% non-Hispanic White people (42.2% vs 32.4, *P* = .008) and among tracts that contained LILA census tracts compared with those without LILA tracts (41.1% vs 30.4%, *P* < .001). In multivariable models, consideration of food retail accessibility in assessment of public transit routes was significantly associated with greater population size, Western (compared with Southern) census region, and greater poverty prevalence.

**Table 2:** Consideration of Access to Retail Food Locations in Assessment of Public Transportation Routes^a^, National Survey of Community-Based Policy and Environmental Supports for Healthy Eating and Active Living, 2014

Municipality Characteristics	% Reporting Yes (95% CI)	χ^2^ *P* Value	Odds Ratio^b^ (95% CI)
**Overall** (n = 1,338)	33.8 (31.3–36.4)	Not Applicable	
**Population^c^**			
<2,500 (n = 381)	16.8 (13.1–20.6)	<.001	1 [Reference]
2,500–49,999 (n = 822)	38.0 (34.6–41.3)		3.2 (2.0–4.9)
≥50,000 (n = 135)	55.9 (47.5–64.3)		5.9 (3.3–10.2)
**Rural/urban status^d^**			
Urban (n = 1,070)	37.1 (34.2–40.0)	<.001	1 [Reference]
Rural (n = 268)	19.6 (14.9–24.4)		1.1 (0.6–1.7)
**Census region**			
Northeast (n = 168)	26.8 (20.1–33.6)	.003	0.8 (0.5–1.3)
Midwest (n = 469)	33.7 (29.5–38.0)		1.2 (0.9–1.7)
South (n = 422)	32.2 (27.8–36.6)		1 [Reference]
West (n = 279)	43.1 (37.3–48.9)		1.4 (1.01–2.0)
**Median educational attainment**			
Some college or more (n = 801)	35.7 (32.4–39.1)	.08	1 [Reference]
High school graduate or less (n = 537)	31.0 (27.1–35.0)		1.0 (0.7–1.3)
**Poverty prevalence^e^**			
<20% (n = 922)	32.2 (29.2–35.3)	.07	1 [Reference]
≥20% (n = 416)	37.4 (32.7–42.0)		1.3 (1.0–1.8)
**Race and ethnicity**			
>50% Non-Hispanic White (n = 1,142)	32.4 (29.7–35.1)	.008	1 [Reference]
≤50% Non-Hispanic White (n = 196)	42.2 (35.3–49.2)		1.2 (0.8–1.7)
**Low-income low-access (LILA) status^f^**			
Contains LILA tracts (n = 462)	41.1 (36.6–45.6)	<.001	1.0 (0.8–1.3)
Does not contain LILA tracts (n = 831)	30.4 (27.2–33.5)		1 [Reference]

a Among municipalities that have public transit.

b Adjusted for population size, rural/urban status, census region, educational attainment, poverty prevalence, and race and ethnicity.

c Among the population aged 25 years or older.

d Rural/urban status was derived from the 2010 US Census Urban Area to Place Relationship File, with municipalities classified as urban if more than 50% of the population resided in areas defined as urban (16).

e Percentage of the population living below federal poverty guidelines.

f Model 1 includes all the municipalities and examines the associations for all the variables except the LILA variable; model 2 is used for the LILA variable and only includes municipalities with that variable.

Results remained similar in sensitivity analyses that excluded municipalities that responded don’t know to the public transit question (n = 165) or the dedicated transportation question (n = 78). With these respondents removed, prevalence of not having public transit was 36.7%, prevalence of dedicated transit to food locations was 15.4%, and 38.7% of municipalities with public transit considered food retail accessibility in route planning. All associations of transportation supports with community characteristics remained significant, except that poverty prevalence was no longer associated with considering food retail accessibility in assessment of public transit route planning.

## Discussion

Findings from our study suggest that planning supports for food access are not common in municipalities with transportation systems in place. Only 1 of 3 municipalities (n = 452) with public transit available reported that they considered food retail accessibility when planning transportation routes. Fewer than 1 in 7 (n = 299) municipalities had alternative options, such as dedicated vans or shuttle buses, to allow residents to easily access food retail locations, and few municipalities without public transit offered DRT options. These findings are consistent with national data for public transit availability (6) and other studies that have documented significant transportation barriers to accessing food (9,21,22). Some studies have examined food accessibility as part of comprehensive planning documents, but to our knowledge none have investigated the prevalence of municipal-level transportation supports for accessing food (23). Providing affordable transit options with direct routes to existing food retail locations, or within a reasonable walking distance from the entrance to the food retail store or farmers market, can improve the accessibility of food, which may facilitate the reduction of health disparities (24) and rates of obesity (8–11).

In our study, the prevalence of transportation supports varied greatly by census region. Presence of DRT was more commonly reported in the Northeast and the less in the South, while the Western census region more commonly reported consideration of food retail accessibility in assessment of public transit routes. The effects of expanding transportation may be especially beneficial in the South for increasing food accessibility, where one-third of adults have obesity, rates of heart disease and diabetes are highest in the US, 12% of the people are living with food insecurity, greater than 20% of the counties are considered to be in persistent poverty, and the area has the highest shares of LILA census tracts (25).

Our study also found significant urban/rural disparities in transportation supports for food accessibility. Rural municipalities were significantly less likely to report presence of public transit, and just 7.9% (n = 43) reported presence of DRT to food retail locations compared with 17.2% (n = 252) of urban municipalities. Although vehicle ownership rates are higher among rural residents than urban residents, 1.3 million (4%) rural households do not own a car and are found predominately among low-income households (5). Rural residents are older, have less access to basic services, higher rates of poverty and obesity, and are more likely to be food insecure and to die earlier from diet-related preventable diseases (25,26). An overall lower availability of transit and policies to support food access in rural areas and communities with smaller populations might be explained by the ways in which transportation is funded, such as requiring municipalities to match funds and determining funding based on population size estimates. Municipal-level budget and priorities are affected by changes in total population, thus even small decreases in population can result in decreased funding for critical services, such as public transportation (13). Municipalities can consider braiding multiple funding streams and collaborating with organizations to explore transit solutions that support food access. For example, if administrative funds are available from the state agency, municipalities can consider partnering with senior centers or area agencies on aging to leverage funds from the state’s Senior Farmers Market Nutrition Program (SFMNP) to fund transportation components that make it easier for seniors to get to the farmers market and redeem SFMNP benefits (24).

DRT options, such as community vans or shuttle buses, are especially important for populations with special needs, such as seniors or persons with disabilities who may have difficulty accessing public transit or be unable to drive themselves. These services are the second largest type of transportation service in the US and the main provider of transportation in rural areas, but account for less than 0.5% of all national passenger trips (6) — although in this study they were more commonly reported among urban municipalities (17.2% urban vs 7.9% rural). Because traditional transit options (eg, fixed bus routes) may not be feasible or practical in rural communities, rural funding commonly supports alternative models (eg, demand responsive) that are cost-effective for low-density areas and better suited to meet community need (12,24). An example is the Living Independence Network Corporation in Twin Falls, Idaho, that provides residents with transportation vouchers for trips such as grocery shopping, medical appointments, recreation, and employment (12). However, our results suggest that few communities without other public transit offer these services.

It is encouraging to see municipalities with at least 1 LILA tract designation considering food access in their transportation planning, compared with those with no LILA tracts (41.1% vs 30.4%). However, among municipalities that considered food access in their transportation planning, the difference between municipalities with LILA tracts and municipalities without LILA tracts did not remain significant after adjusting for other characteristics in multivariate models. Our data suggest that municipalities with designated LILA tracts may be taking concerted steps to address food access issues through transportation planning and prioritization of transit routes to food locations. The effects of living in a LILA area can be compounded by lack of transportation, leaving people reliant on social, familial, and care networks (eg, senior center, faith-based organization) for rides to essential services, or pay out of pocket for ride-shares or other privately owned transportation services (eg, taxi). Financial barriers to transportation may disproportionately affect those who have low or no income; those already living with, or at risk for, food insecurity; and people living in low-income, rural communities, who typically have the farthest distances to travel to access food (27). Successful efforts to improve community food access through affordable transportation have been documented (12,24,28). For example, the publicly run Grocery Bus line in Austin, Texas, was designed to take those from a low-income Latino community lacking sufficient transportation options to supermarkets. The Grocery Bus, developed through collaboration between Austin’s Capital Metro transit system, Austin/Travis County Food Policy Council, community members, and supermarkets, was applauded for its convenience and savings (29) and assimilation into the regular transit system (30). Additionally, municipalities can consider public–private partnerships to create or expand low-cost transportation options. As of February 2019, 38 states, Puerto Rico, and the District of Columbia authorize by statute public–private partnerships for transportation (31). For example, The Dallas Area Rapid Transit (DART) GoLink program provides on-demand shuttles, including rides for essential needs (eg, food); DART expanded this service in 2018 by awarding a contract to Uber Technologies Inc to provide UberPool service as a supplement to DART’s GoLink service in all 13 service areas, inclusive of reduced fare rides in select service areas (32).

This study is subject to limitations. First, data were self-reported and not verified with written municipal-level policy sources and may not represent actual policy or implementation. Second, the person who completed the survey may be unaware of transportation to food supports, especially if they were adopted at a regional rather than municipal level; thus, supports may be underreported. Third, despite weighting to make survey responses nationally representative, these findings may be less generalizable because of a low response rate. Fourth, although questions were cognitively tested and the survey was piloted in 2 states before it was fielded nationally, it is possible that the transportation survey questions were interpreted differently by respondents than anticipated. Specifically, respondents may have reported that they had public transit available when in fact they only offered DRT. Likewise, because the survey question regarding DRT asked respondents to not include public transit options, availability of DRT may be underreported because DRT may exist as components of public transit systems. Nonetheless, our results still support the notion that many US municipalities lack transportation supports for food access.

Local governments can address community needs such as food access and transportation through policy and planning. Our study found wide variation in the presence of municipal-level transportation supports for food access, with differences by municipal-level characteristics. Results suggest opportunities to improve food access by using public transportation supports, especially in communities that have small populations, are rural, and are in the South. Expansion of public transit and DRT and improvements to existing transit systems can provide easier travel to food, which may improve diet quality and reduce chronic disease, especially among disproportionately affected populations.
